# Opti-4TB: A protocol for a prospective cohort study evaluating the performance of new biomarkers for active tuberculosis outcome prediction

**DOI:** 10.3389/fmed.2022.998972

**Published:** 2022-09-14

**Authors:** Olivier Bahuaud, Charlotte Genestet, Jonathan Hoffmann, Oana Dumitrescu, Florence Ader

**Affiliations:** ^1^Département des Maladies Infectieuses et Tropicales, Hospices Civils de Lyon, Lyon, France; ^2^Centre International de Recherche en Infectiologie (CIRI), Inserm U1111, Université Claude Bernard Lyon I, CNRS, UMR5308, École Normale Supérieure de Lyon, Lyon, France; ^3^Hospices Civils de Lyon, Institut des Agents Infectieux, Laboratoire de bactériologie, Lyon, France; ^4^Medical and Scientific Department, Fondation Mérieux, Lyon, France; ^5^Faculté de Médecine, Université Claude Bernard Lyon 1, Lyon, France

**Keywords:** tuberculosis, biomarkers, host-pathogene interaction, treatment monitoring, multi omics analysis, host immune response

## Abstract

**Introduction:**

Tuberculosis (TB) treatment requires the combination of multiple anti-TB drugs during 6 months or more depending on strain drug susceptibility profile. Optimizing the monitoring of anti-TB therapy efficacy is required to provide adequate care and prevent drug resistance emergence. Moreover, accurate monitoring tools are needed for the development of strategies aiming at reducing treatment duration. Opti-4TB is a “proof of concept” study aiming at developing a blood-based monitoring of TB outcome by deciphering host immune signatures associated with latency or disease activity through the combination of “omic” methods. The primary objective is to assess the performances of new biomarkers for TB outcome prediction and to determine specific profiles associated with the outcome of treated TB patients.

**Methods and analysis:**

Opti-4TB is a prospective, single center study including adult patients hospitalized for pulmonary TB. A workflow will be set up to study the immune status of 40 TB patients and 20 controls with latent TB infection. Blood samples will be collected at four timepoints: before treatment initiation (V1), at day 15 (V2), at 2 months (V3) and at 6 months (V4). *Mtb*-specific immune responses will be assessed at each timepoint with three different assays: (1) A whole blood transcriptomic signature assessing the “RISK-6” score; (2) A proteomic signature based on 27 cytokines and chemokines measured in plasma; (3) An immunophenotypic monitoring of circulating T-cell subpopulations using spectral flow cytometry. This in depth characterization of *Mtb*-specific immune response throughout the treatment, correlated with clinical outcomes, will lay the basis for the elaboration of the most basic and universal stage-specific immune signatures associated with latency, active disease and cure.

**Ethics and dissemination:**

Ethical approval has been obtained from the institutional review board (n°69HCL18_0757). Results will be communicated at scientific meetings and submitted for publication in peer-reviewed journals.

**Trial registration number:**

NCT04271397.

## Introduction

Tuberculosis (TB) is a leading cause of death worldwide from infectious disease responsible for approximately 1.5 million deaths yearly (1). Only 5 to 10% of individuals infected with *Mycobacterium tuberculosis* (*Mtb*) will subsequently develop active disease during their lifetime, while the others will have latent TB infection (LTBI). LTBI is detected by interferon (IFN)-γ release assays (IGRAs) such as QuantiFERON-TB (Qiagen®) or T-Spot-TB (Oxford Immunotec®). So far, no test can accurately predict the progression from LTBI to active TB. TB is responsible of a broad spectrum of clinical presentations ranging from minor clinical symptoms to disseminated TB. Lungs are the main disease site accounting for 80% of the cases, although many organs can be affected such as lymph nodes, bones and central nervous system, regardless of lung involvement. TB diagnosis is assessed by the isolation of *Mtb* from patient's specimens either in culture or through molecular methods (2). TB treatment always requires multiple drug regimen administered over a time-period ranging from six to ≥ 18 months, depending on drug susceptibility results. The duration of TB treatment along with related adverse events are the main cause of non-compliance leading to the emergence of multi-drug resistant (MDR)-TB (3).

To date, although sensitivity and specificity are low for predicting failure and relapse, culture conversion of respiratory specimens is still used for treatment efficacy assessment (4, 5). In this context, the World Health Organization (WHO) has adopted in 2015 the “End TB strategy” aiming at reducing the TB-related deaths by 95% and the new contaminations by 90% in 2035 (6). To this end, the WHO encourages the development of new diagnostic and prognostic tools to overcome currently existing tools limitations. The development of these new point-of-care tools should therefore focus on the following objectives:

- to improve the diagnostic sensitivity compared to smear microscopy and sputum culture;
- to improve the detection of progression from LTBI to active TB (incipient TB patients);
- to accelerate detection of drug resistance to properly guide treatment strategy;
- to allow treatment monitoring strategies that provide predictive value for treatment efficacy.

In association with the Foundation for Innovative New Diagnostics (FIND), the WHO has set up a series of target product profiles (TPPs) establishing the criteria that these tools should meet in terms of optimal and minimal performance and operational characteristics (7).

The evaluation of biomarkers of the host immune response that correlate with favorable TB outcome is an active area of research. Recently, Zimmer et al. conducted a systematic review and meta-analysis of studies assessing biomarkers for TB treatment monitoring. They identified four biomarkers (CRP, IL-6, IP-10, and TNFα) exhibiting a significant decrease after eight weeks of treatment in several independent studies highlighting the value of these biomarkers for treatment monitoring (8). Studies based on the combination of commercialized IGRAs with an “in-house” IFN-γ release assay based on heparin-binding hemagglutinin (HBHA) stimulation have shown promising results in differencing LTBI from TB patients on whole blood sample (9, 10). Several studies have also confirmed the pivotal role of T-cells and their effector molecules in *Mtb* clearance (11). Modifications in T-cells profiles have been observed between patients with different TB stages. Notably, variations in abundance and phenotypical changes have been found in *Mtb*-specific peripheral CD4+ and CD8+ T-cells subpopulations, as well as MAIT cells among LTBI, active TB, and cured patients (12–17). Recently, based on an in-depth phenotyping with mass cytometry of peripheral *Mtb*-specific T-cells, our group found a differential profile according to sputum conversion. We observed an under-representation of terminal memory effector CD8+ T-cell subpopulations (T_EMRA_) in patients whose sputum cultures were still *Mtb-*positive at two months of treatment compared to patients whose cultures were *Mtb*-negative (18). Successful mycobacterial clearance is linked to CD8+ T-cell effectors, which in turn require CD4+ T-cell engagement (19). Thus, a global evaluation of the anti-TB immune response based on the study of T-cells phenotypic profiles and effector molecules using a peripheral blood-based approach throughout TB treatment might hold the key to overcome the actual limitations in terms of TB treatment monitoring, efficacy and outcome.

### Rationale

Taking advantage of a range of new whole blood-based methods detecting phenotypical changes of host adaptive immune cells and effector molecules abundance variation, we intend to study their performance in indicating stage-specific profiles associated with TB latency or active disease. We hypothesize that specific detectable immune signatures are associated with LTBI, active TB and cured TB. We will address the question of whether favorable TB disease outcome is associated with immune signature reversion, historically assessed by durable negative smear microscopy and culture conversion.

### Objectives

The primary objective of the Opti-4TB project is to assess candidate biomarkers from TB diagnosis to the end of TB treatment:

1) a whole blood transcriptomic signature;
2) a whole blood proteomic signature;
3) an immunophenotypic signature of circulating T-cells.

### Deliverables

The Opti-4TB protocol is a “proof of concept” study aiming at developing a blood-based monitoring of TB outcome by deciphering specific host immunity signatures associated with latency, disease activity and cure, through the use of “omic” methods. Currently, there is no prognostic tool to guide treatment and optimize the control of resistance emergence, consistent with WHO priority objectives (pillar 3 of the END-TB strategy). Ultimately, the goal will be to test these host-based signatures in a large multicenter prospective clinical study along with historical standard methods (smear microscopy, sputum culture).

## Methods and analysis

### Study design

The Opti-4TB protocol is a single center prospective “proof-of-concept” cohort study of adult patients treated for drug-susceptible (DS) pulmonary TB at the Infectious Disease Department of a 5362 tertiary care university hospital (Lyon, France). Over the past decade, an average of 30 to 50 patients with culture-confirmed TB were diagnosed and treated yearly in the department.

### Setting

Eligible DS pulmonary TB adult hospitalized for receiving treatment will be assessed for inclusion in the Opti-4TB protocol. The recruitment period will be of 36 months and the patient overall follow-up will be of 6 months covering a total period of 42 months. A control group of 20 LTBI participants will be included.

### Participants

**Figure 1:**
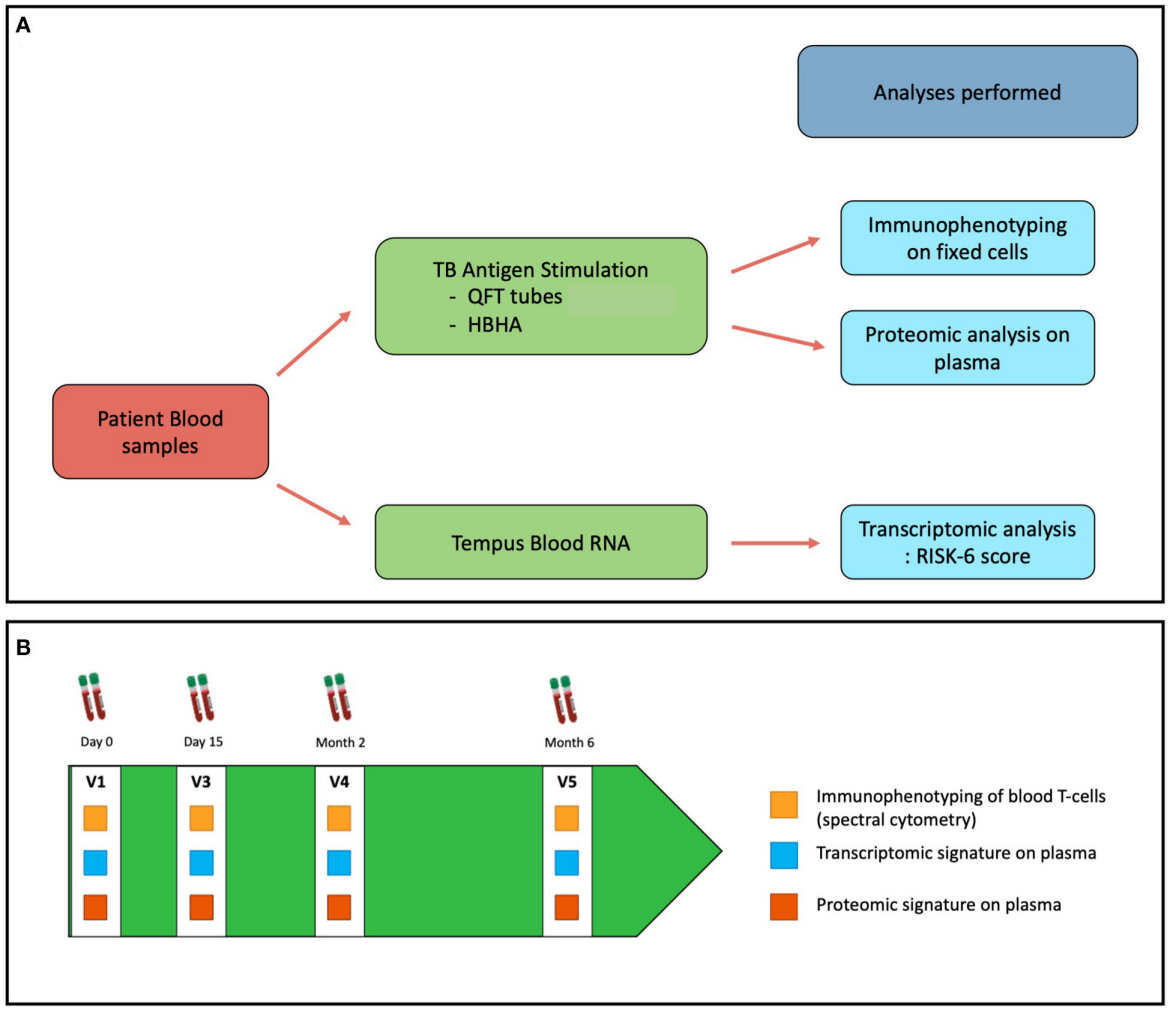
**(A)** Workflow of immune monitoring and **(B)** sampling schedule of the OPTI-4TB protocol.

The workflow of the Opti-4TB protocol is described in Figure 1A. Pulmonary TB patients aged 18 or more will be included. Inclusion and Exclusion criteria are listed in Box 1.

Box 1 **Inclusion and exclusion criteria.**

- **Box 1:**
- Inclusion criteria:
- Adult ≥ 18 year-old
- Patients having given written consent
- Patients accepting a follow up ≥ 6 months
- Proven active pulmonary tuberculosis (positive direct examination and/or PCR)
- Or Latent tuberculosis infection assessed by positive IGRA

Exclusion criteria:

- Malignant solid tumor
- Malignant hemopathy
- Solid organ transplantation or hematopoietic stem cell transplantation
- Immunosuppressive treatments (i.e. biologics, calcineurin inhibitors, corticosteroids)
- Auto-inflammatory disease
- Chronic liver diseases
- Chronic infection with HIV, HCV (hepatitis C virus) or HBV (hepatitis B virus)
- Antimycobacterial treatment initiated > 7 days
- Pregnancy or breastfeeding
- Refusal to participate to the study
- Persons deprived of their liberty by judicial or administrative decision
- Protected adults
- Patients not affiliated to health-care social security
- The homeless

During the first visit (V1), explanations will be given on treatment duration and schedule of blood sampling occurring at the V1, Day 15 (V2), 2 months (V3) and 6 months (V4) visits to monitor treatment efficacy and tolerance. Additional peripheral blood sampling will be collected at each visit for biomarker assessment as described in Figure 1B.

### Endpoints

To address the objectives mentioned above, the primary endpoint will be to evaluate the concordance of the kinetics of the biomarkers with the outcome of treated TB patients at each visit. It is a composite outcome including clinical status, radiological computed tomography (CT)-scan assessment and mycobacterial culture conversion of sputum samples.

This will comprise:

1) A whole blood transcriptomic signature based on levels of expression of a panel of 6 genes;
2) A proteomic signature based on levels of various cytokines and chemokines production measured in plasma;
3) A dynamic phenotypic monitoring of circulating T cell subpopulations through spectral cytometry using a panel of 30 markers;

Secondary endpoints will be to identify profiles, based on the above biomarkers, consistent with three *Mtb* infection stages: LTBI, active TB and cured TB.

### Variables

The following data will be recorded in an Opti-4TB specific electronic Case Report Form (e-CRF): demographics and comorbidities, TB clinical characteristics including symptoms, localization(s) of the lesions, biological and CT-scan findings, anti-TB regimen, and associated therapies. TB microbiological characteristics will include the type of lineage and species of the *M. tuberculosis complex* and the type of sample from which the pathogen has been isolated along with phenotypic and genotypic drug-resistance profile determination. The latter will be obtained through whole genome sequencing (WGS) of each isolate which will also enable the monitoring of *Mtb* micro-diversity (including antibiotic resistance mutations) emergence within each host throughout the treatment.

The PCR-based transcriptomic assay will measure transcripts expression levels of a 6 gene panel named RISK-6 by quantitative real-time (qRT) PCR at each time point (20). The panel includes the following genes: *GBP2, FCGR1B, SERPING1, TUBGCP6, TRMT2A*, and *SDR39U1*. The RISK6 signature score is based on a calculation algorithm taking as input data the cycle thresholds ratio for 9 different pairs of transcripts.

The proteomic assay will measure cytokines and chemokines concentration levels in plasma at each time point using the Bio-Plex Pro™ Human Cytokine 27-plex Assay (Bio-Rad, USA). Cytokines and chemokines will be measured, after whole blood stimulations with either QuantiFERON-Gold Plus kit or an in-House “Heparin-Binding HaemAgglutinin” (HBHA) antigen (21). The concentration levels of IFN-γ measured in this assay will also serve as reference for a double IGRA assay combining the different stimulations mentioned.

The quantitative and functional characteristics of T-cell subpopulations will be assessed by spectral cytometry (Aurora, Cytek® Biosciences, USA) on fixed cells obtained from blood samples taken at each time point and previously stimulated as above-mentioned. A 30 markers panel will be used to identify and quantify each subtype of T-cells. An equal number of total cell events will be recorded to enable comparison between patients and timepoints.

### Biomarker assays

As previously stated, three different assays will be performed to evaluate the performance of immune biomarkers in TB diagnosis and outcome.

For the transcriptomic assay, whole blood will be collected on specific Tempus Blood RNA tubes (Thermo Fischer Scientific, USA) containing a liquid RNA stabilization reagent providing *in situ* stabilization of RNA and transcript profile within the blood samples. These samples will be used to perform qRT-PCR and calculate the RISK-6 score.

For the proteomic and the T-cell phenotyping assays, whole blood collected in heparin tubes (BD Vacutainer® Heparin Tube, BD, USA) will be transferred into QuantiFERON Gold plus kit (QFT-P) tubes for stimulation. QFT-P includes four blood collection tubes: NIL, TB1, TB2, and Mitogen each requiring 1 mL of whole blood. NIL tubes contain no antigens and are used as a negative control. TB1 and TB2 tubes contains the conventional ESAT-6 and CFP-10 antigens, designed to produce cell-mediated immune responses from CD4+ T lymphocytes (22). In addition, TB2 also contains an undisclosed peptide pool that can stimulate both CD4+ and CD8+ T-cells (23). The mitogen tube contains phyto-hemagglutinin used as positive control. An extra 1 mL of whole blood will be stimulated with 10 μg/mL of rmsHBHA. After a 24 h-stimulation (37 °C, 5% CO2 atmosphere) plasma and cells from each of the five conditions will be separated by decantation. Plasma will be stored at −80°C awaiting batch serial cytokine and chemokine quantification with the Bio-Plex Pro™ Human Cytokine 27-plex Assay. Collected cells will be transferred into new tubes and incubated with FACS lysing buffer (Becton Dickinson, USA) to lyse red blood cells and fix all peripheral blood mononuclear cells (PBMCs) and fixed white blood cells pellets will be stored at −80°C until further use. In preparation for flow cytometry, cells will be resuspended and aliquoted for staining. In addition, 100 μL of cell suspension will be aliquoted for unstained controls. A previously described 29-marker panel of antibodies with the addition of a CD19 marker will be used for staining (18). Briefly, the panel contains 28 T-cell oriented surface markers (lineage markers, chemokine receptors, activation markers and exhaustion markers), one intracellular target (perforin) and CD19 to improve resolution (antibodies purchased from BD Bioscience, USA, BioLegend, USA and ThermoFisher Scientific, USA). Cells will be resuspended in Brilliant Stain Buffer (BD Bioscience, USA), stained for 30 min at room temperature in the dark, washed with FACS buffer (PBS 0.1% BSA) and fixed with 4% Formaldehyde (FA) (ThermoFisher Scientific, USA) before intracellular staining in BD PhosFlow Perm/Wash Buffer I (BD Bioscience, USA). Then samples will be fixed for 20 min in freshly reconstituted 1% FA, washed once with PBS, resuspended in FACS buffer, and kept at 4°C until acquisition on Aurora Cytek® spectral analyzer.

### Study sample size

The main objective of this study is the identification of prognosis factors in terms of treatment efficacy. All the variables of interest represent continuous quantitative variables. Moreover, the distributions of the data for the double IGRA and the immunomonitoring assays are quite asymmetrical requiring non-parametric tests for comparison. For these reasons there is no consensus on the number of participants needed. However, the mean sample size of the studies evaluating biomarkers for TB treatment monitoring recently reviewed by Zimmer et al. was of ~50 patients (8). In addition, reference studies assessing T-cell response in TB patients by immunophenotyping such as Adekambi et al. and Ahmed et al. showed significant T-cell phenotypic changes throughout treatment with cohort of 10 and 39 patients respectively (12, 13). Hence, we expect to include 40 patients presenting active pulmonary TB which is consistent with the number of patients diagnosed yearly in the Infectious Diseases department. We consider that it will allow to obtain statistically significant results. The number of 20 LTBI patients was also chosen according to previous studies (13, 24).

### Statistical methods

The prognostic power of each biomarker will be evaluated separately. Biomarkers associated with evolution will be kept for multivariate logistic regression models. These models will allow the obtention of different scores per patient and the characteristics of a composite test will be assessed by studying ROC (receiver operating characteristic) curves and calculating AUC (area under the curve). Analysis of the ROC curves will allow estimation of sensitivity, specificity and AUC via mean and confidence interval obtained by bootstraps.

We will study the kinetics of the different biomarkers (clinical, transcriptomic, proteic, or phenotypic) to identify the most basic and universal immune signatures associated with different TB stages: LTBI, active TB and cure.

When required, analyses will be based on two-sided p-values, with statistical significance defined by p < 0.05 and conducted with the software R. Finally, considering multivariate analyses, appropriate multiple imputations will be taken into consideration in case of relevant amount of missing values.

## Discussion

The strength of the study is to perform a prospective, multiparametric monitoring of patients treated for pulmonary DS-TB. The clinical, microbiological and imagery follow-up performed at four time-points from diagnosis to the end of treatment will ensure consistent data collection. High-dimensional proteomic and transcriptomic analyses have become approachable and have brought new research frameworks and perspectives. The results obtained from “omics” methods will be analyzed according to patient's outcomes. Assuming that these results will provide an in-depth view of *Mtb*-specific host immune response at different stages of TB management (from diagnosis to cure), a step further will be to elaborate the most basic universal “omics” signatures associated with latency, active disease and cure.

The cellular immune response to *Mtb* is intrinsically multifaceted, and further complexified by mycobacterial mechanisms of immune evasion, *Mtb* micro-diversity within each host (25), inter-individual immune heterogeneity. The number of molecular markers and of immune cell phenotypes involved in *Mtb* infection control and of interest for monitoring keeps expanding as our knowledge of the disease progresses. To understand how all these separated insights relate to each other and are connected at the cellular and molecular levels during TB, a comprehensive, deeper profiling of the immune system in relation with TB stages is needed. Consistently, the use of novel spectral flow cytometry, which enables single cell analysis of the expression of surface, cytoplasmic, and nucleic markers using panels of up to 40 markers, is an indispensable tool for understanding the role of T-cell subpopulations across the spectrum of TB disease.

A limit of the study is the “off-site” exploration of host immune profile. The multi-omics evaluation will be performed on peripheral blood samples, whereas the main and primary site of infectious is in the lungs. Thus, it is possible that not all the observed proteomic, transcriptomic and phenotypic changes over time are strictly related to *Mtb*-specific response. However, similarities found in high-dimensional peripheral profiles from one patient to another may easily help discriminate *Mtb*-driven changes. This limitation is linked to the ≪ bench to bedside ≫ approach of the study. It reflects the practical issue of sample accessibility in TB management. We also acknowledge that the important number of variables collected could lead to a lack of statistical power regarding the study sample size. However, a multicenter cohort study will follow this proof-of-concept pilot study to validate the basic universal signature approach.

## Dissemination

Results will be communicated at scientific meetings and submitted for publication in peer-reviewed journals.

## Ethics statement

This study involving human participants was reviewed and approved by French National Review Board for Biomedical Research in May 2019 (Comité de Protection des Personnes Ile de France X) under the title Opti-4TB-69HCL18_0757. The patients/participants provided their written informed consent to participate in this study.

## Lyon TB Study Group collaborators

F. Ader, O. Bahuaud, R. Bayaa, A. Becker, E. Braun, P. Chabert, P. Chauvelot, C. Chedid, A. Conrad, O. Dumitrescu, T. Ferry, C. Genestet, S. Goutelle, E. Hodille, J. Hoffmann, C. Javaux, G. Lina, C. Pouderoux, T. Perpoint, S. Roux, M. Simon, F. Valour.

## Author contributions

OD and FA initiated the project and designed the trial. OB and FA drafted the manuscript and all authors (CG, JH, and OD) were involved in critical revision of the article for important intellectual content and approved the final version. All authors contributed to the article and approved the submitted version.

## Funding

This study was being supported by a grant from the French National Research Agency (Agence Nationale pour la Recherche, ANR) (project PRIM-TB, ANR-18-CE17-0020).

## Conflict of interest

The authors declare that the research was conducted in the absence of any commercial or financial relationships that could be construed as a potential conflict of interest.

## Publisher's note

All claims expressed in this article are solely those of the authors and do not necessarily represent those of their affiliated organizations, or those of the publisher, the editors and the reviewers. Any product that may be evaluated in this article, or claim that may be made by its manufacturer, is not guaranteed or endorsed by the publisher.
